# Late modern war and the *geos*: The ecological ‘beforemaths’ of advanced military technologies

**DOI:** 10.1177/09670106241265636

**Published:** 2024-10-18

**Authors:** Mark Griffiths, Kali Rubaii

**Affiliations:** Newcastle University, UK; Purdue University, USA

**Keywords:** DRC, environment, Gaza, *geos*, Iraq, late modern war

## Abstract

This article develops the idea that late modern war’s relationship with the *geos* (the ground and the life it sustains) is doubly destructive. While part of this is recognized in a recent focus on slow violence and ecological aftermaths, there is little consideration of the ‘beforemath’, or the sites of extraction that make advanced military technologies possible. Drawing attention to mining in the Democratic Republic of the Congo (DRC), the article connects military technologies to arms manufacturers and their use of extracted minerals (e.g. cobalt, tantalum, copper, uranium). Shared patterns of environmental and public health effects across parts of Iraq, Gaza and the DRC indicate the doubly destructive nature of late modern war’s relationship with the *geos*: toxic materials threaten life *after* war as the deposits of bombardment and *before* war as mineral commodities at the beginning of arms supply chains. The article explicates how a perspective from the beforemath radically refigures the ways we think about war and spatiality, temporality, and the range of bodies affected in ways that promise a fuller understanding of the violence distributed by practices of late modern war.

## Introduction

Technological advancements equip militaries with increasingly sophisticated – and ‘precise’ – techniques and munitions that enable a more contained form of violence. Precision targeting kills in an instant, in a predetermined and concentrated blast radius that significantly reduces ‘collateral’ damage. In this prominent and defining aspect of ‘late modern war’, targets are located and eliminated with maximum efficiency, a swiftly open-and-shut mode of distributing violence (Gregory, 2011; Jones, 2020). A suite of bespoke munitions (Bell, 2008; Joronen, 2016), data aggregation software (Wilcox, 2015), GPS/GIS infrastructures (Beck, 2003; Kaplan, 2006) and aerial hardware (Agius, 2017; Borg, 2021) has become essential to this end, enabling advanced militaries (e.g. those of the US, UK, Israel, coalition states) to conduct war in territories deemed ‘hostile’ (e.g. Afghanistan, Iraq, Palestine). At these prominent sites of late modern war, more accurate methods of killing base claims around a reduction of ‘excess’ or ‘uncontrollable’ violence (i.e. ‘collateral’). It is by now a prominent and normalized discourse of politicians, militaries and arms manufacturers to foreground low (or no) collateral as a signifier of the more discerning practices that constitute late modern war. In broader terms, late modern war is marked by emphases on preemption and acceleration (Graham, 2010; Massumi, 2015), ‘humane’ or humanitarian intervention (Gregory, 2010; Moyn, 2022), and (recently) a green agenda that promises a more environmentally friendly way of doing war (Bigger and Neimark, 2017; Harris, 2015). Technological advancements, the argument goes, afford militaries greater discrimination, dramatically reducing excess violence, and thus effecting surer limits on the time-spaces of war and its effects.

In reality, of course, military claims of temporally and spatially contained violence prove insubstantial as a robust body of evidence grows around the ways that late modern practices of war are decidedly *im*precise. In the longue durée of war’s aftermaths in, for example, Afghanistan, Gaza^1^ and Iraq, already vulnerable ecologies and populations are exposed to the toxic legacies of military operations: weapons-grade heavy metals contaminate farmlands and aquifers (Fathi et al., 2013; PENGON, 2015), military burn pits threaten pulmonary conditions (Rubaii, 2020), and the residues of precision munitions correlate with genetic disorders (Alaani et al., 2020; Manduca et al., 2017). The cumulative charge from study of these effects of war is that the precise weaponry used by advanced militaries causes less readily visible forms of longer-term harm. If weapons residues and military detritus cause congenital disorders, as is widely reported,^2^ there is therefore a need to reconceptualize our understandings of contemporary military violence. Seen from the perspective of those who live in targeted territories, military violence is anything but contained; it instead resides in and issues from the Earth’s constitutive and *militarily instrumentalized* elements – soil, water, air – presenting a protracted threat to ecological and human health. A reconceptualization of war’s effects that recognizes this enduring dynamic has taken form in recent years as critical scholars have sought to break apart the temporal and spatial ontological bounds of military violence (see e.g. Griffiths, 2022; Griffiths and Redwood, 2024; Nixon, 2011; Pugliese, 2020). From this we learn the crucial lesson that, contrary to the justifying logics of precision, military violence in this late modern period is in fact disperse, enduring, indiscriminate.

In this article we begin from the base observation that attending to ‘slow violence’ and the toxic legacies of war forms an additional binding ontological assumption: that war’s relationship to the Earth and the life it sustains (or to use the term we expand on below, *geos*) emerges only in *consequence.* That is, in the important critical work centred on ‘postbellum ecologies’ (Pugliese, 2020) or war’s ‘ecological aftermaths’ (Nixon, 2011), focus is drawn solely on the *fallout* of war. Far less attention is given to the provenance of the materiality that is eventually – *after* the processes of mining and deployment – redeposited into the *geos* to such delayed and devastating effect. If heavy metals such as tantalum, cobalt and uranium contaminate *post*-conflict landscapes, and thus signal a destructive relationship between war and *geos*, then an extended enquiry must consider the sources of such (and other) materials and that *geos–*war relations might therefore further disperse (see e.g. Voyles, 2015). Based on this preliminary observation, we forward here the idea that late modern war’s relationship with the *geos* (the ground and the life it sustains) is *doubly* destructive: the Earth is mined for raw materials that are processed, packaged and reintroduced into the ground, where they leach and seep to become newly threatening to life. The *geos* is thus brought into the violent ends of war in two key ways: first, in the provision of materials that are refashioned to effect harm; and second, in the harbouring of those same materials with a capacity to gradually deteriorate life. While the latter is subject of a burgeoning research agenda on ‘slow’ and cognate registers of violence – e.g. ‘enduring violence’ (Jones, 2022), ‘slow wounding’ (Joronen, 2021), ‘toxic violence’ (Logan, 2018) – the former remains under-investigated and under-theorized. A key question in the age of advanced military technologies is of the increased extractive burden placed on the Earth, where demand for the materials that make electronics possible signals an integral *geos*–war relationship that *precedes* military operation. It is this ‘beforemath’ that makes the toxic aftermath possible, and we know very little of it.

Towards building knowledge of this originary scene of late modern war, the article proceeds in four sections. In the first we present an overview of existing perspectives on war, violence and time with specific focus on a recent turn to ‘ecological aftermaths’ to formulate our founding question: if materials of the Earth that make up precision weapons have repercussions *after* war, then what are their relations to environmental and bodily harm before deployment? In the second section we begin our response by journeying from the aftermath to war’s origins via a survey of military hardware used in two specific contexts – the 2003 US-led invasion of Iraq and Israel’s 2008–9 ‘Operation Cast Lead’ in Gaza – that indicates the sites of extraction that make war possible. The third section considers evidence from the Democratic Republic of the Congo (DRC), where technologically critical elements are mined, to begin a fuller examination of the contingencies between different sites of war, and to crucially argue that the violence of extraction articulates with the violence of war. In the fourth section we argue that a perspective from the ‘beforemath’ radically refigures the ways we think about war and spatiality (from aeriality to subterranean), temporality (from acceleration to delayed or gradual violence) and the range of bodies affected (to include those exposed to the harm of extraction). Only by attending to these refigured bounds of key parameters, we argue in conclusion, can we more fully understand and expose the vast scales of violence germane to the era of late modern war.

## War, violence, time

With important precedents, over the last decade or so scholars have developed understandings around the enduring environmental and health effects of contemporary warfare. Concepts such as ‘toxic aftermaths’ and ‘slow violence’ have gained purchase in examination of recent wars in Afghanistan and Iraq that picks up a line of enquiry on the environmental destruction that marked the era of postwar nuclear testing and the deployment of ecological weapons such as Agent Orange (see e.g. Austin and Bruch, 2000; Martini, 2012). While we do not wish to draw too neat a line between historical and contemporary cases of war’s environmental effects, our interests here are concentrated not on materialities of war that are indiscriminate or ecological by design (i.e. atomic or herbicidal), but on those that are *precisely* the opposite: the discriminating means of violence that is constitutive of ‘late modern war’. By this term we refer to a technology-driven way of war enabled by ‘advanced systems of sensing and surveillance from air and space platforms . . . and weapons systems revolving around pilotless aircraft, robotic vehicles and precision-guided weapons’ (Gregory, 2010, 160; see also Jabri, 2016). Late modern war thus serves as useful shorthand for a shift in strategy and technological capacity since the First Iraq War (1990–1991) that centres on a principle of precision via surveillance- and intelligence-led operations. An important body of literature takes an approach of explicating the details of such operations – e.g. drone piloting (Gregory, 2011), surveillance ‘pattern of life’ data (Wilcox, 2015), the prominence of ‘war lawyers’ (Jones, 2020) and (over)claims on weapon accuracy (Zehfuss, 2011) – and critiquing this supposedly more ‘humane’ (Moyn, 2022) mode of warfare as one that in fact renders certain lives more readily disposable (Agius, 2017; Kaplan, 2017). The technologies and claims of precision that characterize late modern war are thus made the object of study in terms of veracity, ethics and underlying logics to do with a racialized distribution of valued and devalued life.

A focus on the toxic aftermaths of war forms a complementing area of critique that shifts emphasis from accelerated and technologized forms of violence onto the longer-term harm effected by advanced military technologies. Perhaps the most established work of this type is Nixon’s (2011: 205) *Slow Violence and the Environmentalism of the Poor*, whose focus on ‘ecologies of the aftermath’ draws attention to ‘a new fatal kind of environmental imprecision to “precision” warfare’. Centred mostly on the context of Iraq and the US-led coalition’s deployment of depleted uranium munitions, Nixon (2011: 219) weighs evidence of ‘the uranium-compromised aftermath’ using examples of returning servicepeople to highlight the potential dangers of postwar ecologies:

One man’s [sic] precision-guided missile is another man’s weapon of indiscriminate destruction. With depleted uranium, we’re not talking about rogue missiles that accidentally shred a marketplace or a wedding party. We’re talking about the triumphant, pinpoint strike that doubles as a chaotic weapon, a weapon that haphazardly strikes down civilians who, whether under some future tyranny or future democracy, just happen to live downwind in time. (Nixon, 2011: 220)

Similarly focused on the temporal ‘downwind’, Joseph Pugliese (2020: 100) concentrates on Gaza’s ‘postbellum ecologies of the aftermath’ in which the living body (human and non-human) is subject to ‘attritional violence, its [the body’s] physiological processes of ingestion (of contaminated food and water), inhalation (of polluted air) and percutaneous absorption (of heavy metals such as lead) become inscribed with pathogenic and necropolitical effects’. For Pugliese, a ‘forensic ecology’ that traces these processes connects Israel’s acts of war with a deeply embedded structure of violence that extends through Gazan time-spaces. Work that recognizes and interrogates such temporal and spatial extensions of war includes discussion of public health and military burn pits in Iraq (Rubaii, 2020), the effects on non-human species outside of ‘recognisable times of war’ (Leep, 2022: 8), the enduring violence of war-inflicted wounding (Jones, 2022), the politics of knowledge production on militarized toxicity in Iraq and Afghanistan (Logan, 2018; The Nation, 2020), and the ‘geontological’ dissolution of life/non-life binaries in the aftermaths of ‘precision’ warfare (Griffiths, 2022). Late modern war from the angles presented across this research is anything but precise; it is a multitemporal mode of violence that ranges from the spectacular and kinetic to the drawn-out and delayed – war’s *end* is but a ‘mirage’ (Nixon, 2011: 207).

As we discuss further in the following section, a large amount of medical, epidemiological and agricultural research adds weight to such a claim. Medical researchers at key sites of recent wars – Basra, Fallujah, Gaza City – have documented the ways that the ‘massive use of unconventional [technologically advanced] weapons’ leaves residues to ‘percolate slowly into the aquifer, adding pollutants such as cadmium, copper and lead [that] pose serious health risks including cancer’ (PENGON, 2015: 19, 35). The research in these areas forms a mature body of literature, but there are similar patterns at sites where researcher access and infrastructure are severely constrained (on Afghanistan see BBC, 2008) and/or where war is ongoing (for preliminary reflections on Gaza see Qumsiyeh, 2024; on Ukraine see Kireitseva et al., 2023). The signs are telling: there are longer-term effects of the so-called precision capabilities that currently define wars in these areas. This teaches us that the time-spaces of late modern war are not so much compressed as expansive and that a *geos–*war relationship is thus significantly protracted. Crucially, however, this increasingly recognized expansive nature of war is pronouncedly skewed towards the *aftermath* and current research is thus orientated around the question of what happens *after* war? This is a key question that we do not wish to detract from, but there is a corollary written into the literatures reviewed in this section. If materials of the Earth that make up precision weapons – e.g. white phosphorus, depleted uranium and other heavy metals (cobalt, lead, tantalum, tungsten and so forth) – have repercussions *after* war, where do they come from and what are their relations to environmental and bodily harm *before* deployment? How, then, is war’s relationship to the *geos* doubly destructive? From here, our objective is set out clearly: just as recent work on contexts of late modern war has questioned ‘the mirage of war’s end’ (Nixon, 2011: 27), we wish to focus similarly on its illusory beginning. All of those materials are drawn from the Earth; in tracking provenance, we come closer to war’s beginning in the *geos.* It is to this task that the next section turns.

## War technologies as extracted *geos*

In this section we work towards the origins of war via two specific settings: the US-led invasion of Iraq in 2003 and Israel’s ‘Operation Cast Lead’ bombardment of Gaza between 2008 and 2009. This narrowed focus enables greater detail of the practices of late modern war and examination of how they relate to the *geos*; resetting the cases into the wider contexts of protracted war – as we do at the article’s conclusion – reveals something of the scale of the effects we discuss here. We learn that the *geos* is not only the site of toxic aftermaths, it is the spatial and temporal origin of war’s violence.

The arsenal used during the US-led invasion of Iraq (19 March–18 April 2003) was vast in scale: coalition air forces flew 41,404 sorties, dropping a total of 19,040 precision-guided bombs, 8885 unguided unitary munitions and 1276 cluster bombs (HRW, 2003). Table 1 details these munitions and the aircraft used. Subsequent investigations have found that in addition to (or as part of) the weapons listed in Table 1, 116,000kg of depleted uranium was expended during the invasion (Zwijnenburg and Weir, 2016). A total of 7269 Iraqi civilians and somewhere between 13,000 and 45,000 Iraqi military personnel were killed in the assault.^3^ There is also a catalogue of longer-term repercussions to do with depleted healthcare services (Al Hilfi et al., 2013), elevated levels of pollution (Al-Shammari, 2016) and a high prevalence of psychological trauma (Crawford, 2013: 17) that evidence the wider effects of war. Additionally, medics in Iraq have documented connections between weapons remnants and different health effects. For instance, a 17-fold increase in congenital disorders in the cities of Basra and Fallujah is linked by doctors to weapons residue exposure and higher-than-normal (5–6 times) levels of heavy metals (lead, mercury, uranium) in the built environment (Al-Sabbak et al., 2012). Another team of doctors in Fallujah has concluded that ‘the metal load of *Fallujies* in general is unusually high for metals associated with weaponry’ and that this ‘can condition differently MS [miscarriage] and BD [birth defects]’ (Alaani et al., 2020: 8). Oncologists in Basra have attributed high levels of uranium in leukaemia patients to the fact that ‘Basrah is the region which received the highest amount of DU [depleted uranium] during the Gulf Wars’ (Al-Hamzawi et al., 2014: 1271) and agronomists in Mosul have examined how depleted uranium deposits carve new ‘pollution pathways’ that ‘may have serious impacts on the regions’ food chains and subsequently on human health across Iraq: largely through plant uptake and edible food crops’ (Fathi et al., 2013: 7).

**Table 1.** Breakdown of military hardware used in the 2003 invasion of Iraq (adapted from HRW [2003]). Aircraft

Aircraft	Number	Sorties
*Fighters*	735	20,228
*Bombers*	51	505
*Other (including drones)*	1015	20,671
***Total***	**1801**	**41,404**

**Table:** Precision-guided munitions

Designation	Name or nickname	Type	Number
*BGM-109*	Tomahawk land attack missile (TLAM)	Cruise missile	802
*AGM-114*	Hellfire	Laser-guided missile	562
*AGM-130*	Television- or infrared-guided missile		4
*AGM-65*	Maverick	Television-, infrared-, or laser-guided missile	918
*AGM-84*	Stand off land attack missile-extended response (SLAM(ER))	Cruise missile	3
*AGM-86C/D*	Conventional air-launched cruise missile (CALCM)	Cruise missile	153
*AGM-88*	High-speed anti-radiation missile (HARM)	Anti-radar missile	408
*AGM-154*	Joint stand off weapon (JSOW)	GPS/inertial navigation system-guided glide missile	253
*EGBU-27*	Penetrator	Laser- and GPS-guided missile (2000 lb)	98
*GBU-10*	Paveway II	Laser-guided bomb (2000 lb)	236
*GBU-12*	Paveway II	Laser-guided bomb (500 lb)	7114
*GBU-16*	Paveway II	Laser-guided bomb (1000 lb)	1233
*GBU-24*	Paveway III	Laser-guided bomb (2000 lb)	23
*GBU-27*	Penetrator	Laser-guided bomb (2000 lb)	11
*GBU-28*	Bunker buster	Laser-guided bomb (5000 lb)	1
*GBU-31*	Joint direct attack munition (JDAM)	GPS-guided bomb (2000 lb)	5086
*GBU-32*	JDAM	GPS-guided bomb (1000 lb)	768
*GBU-35*	JDAM	GPS-guided bomb (1000 lb)	675
*GBU-37*	JDAM	GPS-guided bomb (5000 lb)	13
*Various*	UK guided munitions		679
***Total***	**19,040**		

**Table:** Unguided munitions.

Designation	Type	Number
*M117*	General purpose bomb (750 lb)	1625
*Mk-82*	General purpose bomb (500 lb)	5504
*Mk-83*	General purpose bomb (1000 lb)	1692
*Mk-84*	General purpose bomb (2000 lb)	6
*Various*	UK general purpose bombs	58
***Total***	**8885**	

**Table:** Cluster bombs.

Designation	Name	Guidance	Number	Submunitions/ weapon	Total submunitions
*CBU-87*	Unguided	118	202	23,836	
*CBU-99*	Rockeye	Unguided	182	247	44,954
*CBU-103*	WCMD	818	202	165,236	
*CBU-105*	Sensor fuzed weapon	WCMD, Infrared	88	40	3520
*CBU-107*	Passive attack weapon system	WCMD	Non-explosive		
*UK RBL-755*	Unguided	70	147	10,290	
***Total***	**1276**	**247,836**			

During Operation Cast Lead (OCL) (December 2008–January 2009), the Israeli Defense Forces (IDF) conducted 1418 air strikes hitting more than 1109 targets in Gaza,^4^ as well as a significant ground campaign and shelling from offshore. Around 5400 bombs and more than 7000 artillery rounds were discharged, as well as ‘special weapons’ such as tungsten bombs, white phosphorous and depleted uranium.^5^ Table 2 is extracted from a *Journal of Palestine Studies* report which itself was compiled using a wide range of news, NGO and human rights investigations into OCL (Esposito, 2009a, 2009b). The table details the military hardware used in the assault and some indication of the types and quantities of munitions dropped on the territory. In common with the invasion of Iraq, it is immediately clear that the explosive violence visited on Gaza was massive. More than 1400 Palestinians were killed and at least 5300 were injured during the 22 days of bombardment, around 11,000 homes were damaged or destroyed, and the total cost of repairs to infrastructure was estimated at US$2bn (Al-Haq, 2009). There were many further repercussions to do with wounds and access to healthcare (Jones, 2022), higher levels of pollution (OHCHR, 2009) and psychological trauma (Llosa et al., 2012) that are each part of the less visible effects of war. Over a longer (and ongoing) period of time, medical researchers in Gaza have additionally documented connections between the material remnants of OCL and a range of genetic and reproductive health effects. For example, epidemiological research by a team of doctors at Al-Shifa hospital in Gaza City identifies a ‘causative/favoring role of acute exposure of parents to weapons-associated contaminants . . . on the embryonic development of their children’ (Naim et al., 2012: 1732, 1733) formed through teratogen and toxicant metals that are ‘delivered by weaponry’ (Manduca et al., 2014: 5209). Studies into specific weapons thought to be used in OCL – such as so-called ‘weapons without fragments’ or DIME (dense inert metal explosive) – demonstrate the presence of toxic and carcinogenic metals in the fragment-free wounds they produce that, doctors advise, ‘carries unknown long-term risks for survivors’ (Skaik et al., 2010: 1).

**Table 2.** Military hardware used during Operation Cast Lead (adapted from Esposito [2009a, 2009b]).

	Equipment	Munitions / surveillance hardware
***Planes***	F-16 fighter aircraft	M-82 (500 lb) (many fitted with Paveway II or JDAM precision guidance upgrade kits)
		M-84 (2000 lb)
		PB500A1 (1000 lb Israeli variant of M-83)
		GBU-39
***Helicopters***	AH-64 Apache attack helicopter	M230 cannon
		AGM-114 Hellfire (45 kg)
		Hydra 70 (can be fitted to carry white phosphorous)
	AH-1F Cobra	TOW 2 (or one of the two copies of TOW manufactured in Israel, the Mapatz and the Orlev)
***Artillery***	Soltam M-71 towed howitzer	M433 40 mm HEDP high explosive cartridges
	M109 self-propelled howitzer	M889A1 81 mm high explosive
	Sholef 155 mm self-propelled howitzer	M107 155 mm (designed to spray ~2000 pieces of shrapnel)
	M270 MLRS multiple rocket launcher	M141 83 mm bunker defeat munition
		M930 120 illuminating cartridges
		M971 120 dual-purpose cluster bomb containing 24 submunitions, each containing 1200 fragments
***Tanks***	Merkava tanks (II, III, IV)	105 mm or 120 mm laser-guided anti-tank missiles
		7.62 mm or 12.7 mm heavy machine guns
		60 mm mortars
		Smoke grenades
		RPG-7 (grenade launcher carried by soldiers accompanying tanks)
		M72 LAW (anti-tank weapon)
		B300 Shoulder-launched multipurpose Assault Weapon
***Armoured personnel carrier***	M11 (or Nagmash)	
***Armoured bulldozer***	CAT D9	
***Drones***	Hermes 450	Surveillance (COMINT and ELINT)
		AGM-114 Hellfire
	Heron	Surveillance (COMINT and ELINT)
	Searcher 2	Surveillance (COMINT and ELINT)
	MQ-1 Predator	ISTAR (intelligence, surveillance target acquisition and reconnaissance equipment)
		AGM-114 Hellfire
***Blimp***		ISTAR (tethered over Erez border crossing)
***Sea craft***	Super Dvora-class patrol boats	Unspecified but capacity for surface-to-surface missiles (AGM-114 Hellfire) and light artillery
***Special weapons***		GBU-28 (630 lb bunker buster)
		GBU-39 (250 lb)
		Spike anti-armour missile
		APAM anti-personnel rounds
		MATADOR anti-armour weapon
		White phosphorous
		DIME (dense inert metal explosives)
		Depleted uranium
		Flechette
		Roof knocking ‘fakes’
		Mini cue shrapnel

The longer-term effects documented in both Iraq and Gaza disclose a contingent relationship between war’s materiality, the military instrumentalization of Earth’s constitutive minerals, and the body’s vulnerability to ecological change. It is in this contingency that the ‘slow violence’ of war consists, in the body’s dependence on a *geos* subjected to the interventions of war. In the bombed-out landscapes of Iraq and Gaza, an admixture of weapons-grade heavy metals poses grave threat to current and future generations, depleting the conditions necessary for healthy life. The violence of war in this respect is owed, at base, to the displacement of the *geos*, to the mining, processing and redepositing of the Earth’s elements from and back into the Earth’s crust. Taking this perspective on the assemblage of hardware detailed in Tables 1 and 2 reveals something of the quality of this relationship. Powered by millions of litres of distilled petroleum, thousands of tonnes of iron, aluminium and titanium fuselages deliver lethal packages of heavy and/or radioactive metals in bomb form. Put this way, the *geos*–war relationship is profound: war begins and ends in the Earth – and the depth of the connection is still only the subject of emerging research (Belcher et al., 2020; Crawford, 2022).^6^ It is both an obvious and underarticulated point that late modern war depends on the mobilization of the *geos* to deadly ends, and that this might (or *should*) significantly reshape the ways we know war. In the most generic sense, late modern forms of war depend entirely on elements of the Earth’s crust: metals such as copper (wiring), nickel-chromium (resistors), lead and tin (solder), as well as the crucial metalloids silicon and germanium (for transistors). Without these minerals there would be no electronics in military hardware (or anywhere).

In more specific terms, these and other technologically critical elements are volumetrically minor yet functionally essential to the military assaults of Iraq and Gaza. Cobalt, for example, is used in F-16s for heat exchanger alloys to enable greater functionality at higher temperatures,^7^ a metallic anti-radar coating of the aircraft body,^8^ and in the (DARPA-developed) ‘rare-earth permanent magnet’ that tracks and diverts anti-aircraft weapons.^9^ Cobalt disulphide, in addition, is a commonly used cathode for the high-performance thermal batteries of the type that power the Paveway and joint direct attack munition (JDAM) precision systems of TOW and Hellfire missiles, both of which were used in Iraq and Gaza. Tantalum was also crucial to both operations for its use in semiconductors in high-performance electronics. Stop the War Coalition (2013) claims that ‘the basic circuitry in drones is built with tantalum from refined coltan’ and ‘at the current rate, the weapons industry could exceed smartphone and tablet makers in coltan consumption if it has not already’. The ~50 drones that took part in the invasion of Iraq were considered by the Pentagon as ‘absolutely critical to the speed and scope with which the coalition was able to press the attack’^10^ and the 12 drones that were over Gaza at all times during OCL (Esposito, 2009b: 176) identified targets for F-16 strikes and carried out strikes directly, killing at least 48 Palestinian civilians (HRW, 2003: 3). In addition to keeping drones aerial, tantalum is a key ingredient of the infrared camera tubes in night vision equipment,^11^ for example in the litening pods carried by aircraft such as the US B-52 and Israeli Hermes 450 drone.^12^ Litening ‘is an electro-optical infrared sensor system [that enables] . . . precision targeting, close air support, intelligence, surveillance and reconnaissance’.^13^ The significance of this military capability – owed to the adsorbent qualities of tantalum – should not be understated: ‘night vision devices . . . enhanced lethality and situational awareness in reduced visibility’ for the US Marine Corps in Iraq;^14^ and during OCL ‘the IDF attacked mostly at night in order to take advantage of the lack of night-vision capability among Hamas forces’ (Johnson, 2011: 117).

It is not simply that ‘newer’ metals have enabled this mode of war, there are also minerals with long histories of human use that have taken on different roles in war technologies. Copper and its alloyed forms, for example, have long been used in early metallurgic war (as bronze) and as ammunition casing (as brass). In recent wars, copper has multiple other uses: copper grease prevents heat damage around F-16 gunports;^15^ copper cabling channels communications, such as with the surveillance blimp tethered over Erez crossing during OCL;^16^ and fine copper wires guide TOW missiles to their targets. Copper’s main importance in this era of high-tech warfare, however, is as the basis of all electronics circuitry; none of the hardware listed in Tables 1 and 2 would function without its conductive properties. Another metal newly purposed for war is uranium, which is used in depleted form (isotopes U-235 and U-234) for armour plating (e.g. on tanks) and in armour-piercing ammunition. Its use is controversial: a UN Sub-Commission on Human Rights (UN, 2003) has labelled depleted uranium a ‘weapon of indiscriminate effect’ and there is a vast and contested literature produced by a range of institutions (e.g. RAND, 2005; Royal Society, 2002) and from differing scholarly perspectives (e.g. Bleise et al., 2003; Fairlie, 2009). There is little military-produced literature on depleted uranium available, but it is known that the reasons for its deployment consist in the mechanics of high-energy collisions. Once alloyed with titanium, its high density (twice that of lead) allows for a smaller volume that vaporizes on impact, its pyrophoric properties ‘turn[ing] the inside of a vehicle into an inferno of white-hot gas and sparks’ (Hambling, 2000). In the years following the invasion of Iraq (where depleted uranium was used extensively) and OCL (where its use is suspected), a new generation of armour-piercing ‘SADARM’ (seek and destroy armour) weapons has been developed, of which 121 experimental-form rounds were used in Iraq (HRW, 2003). There is little public information on SADARM save that its main heavy metal component is tantalum.^17^

This is a brief survey of the extensive arsenals of military hardware used in Iraq and Gaza, and it is important to emphasize that cobalt, tantalum, copper and uranium are just four minerals of many. A European Commission (2016) report into ‘raw materials in the European defence industry’ identifies 39 different raw materials and 47 alloys that are crucial for military deployment, and the United States Department of Defense (2021: 158, 159) lists 58 ‘non-fuel commodity’ imports it relies on ‘to maintain technical dominance over adversaries’. Towards a deeper understanding of war’s relationship with the *geos*, this opens the issue to a great many sites of extraction and invites questions around how ‘commodities’ [minerals] are sourced and under what conditions. Two obvious obstacles stand in the way of such enquiry: militaries and arms producers do not volunteer information on products and composition, especially on the toxicity of munitions (Zwijnenburg and Weir, 2016); and raw material supply chains are notoriously convoluted, making geological provenance difficult to determine – an indeterminacy that allows ‘downstream’ manufacturers a stance of plausible deniability (Smith, 2021: 269–207). Despite a ‘mystique of traceability’ (Smith, 2021: 269), recent legislation such as the EU Conflict Minerals Regulation (2021) and the US Dodd Frank Act (2010) generates documents that give important clues. The Dodd Frank Act, for example, requires manufacturers that use technologically critical minerals to submit reports to the Securities and Exchange Commission. ‘Gold and columbite-tantalite (coltan), cassiterite, wolframite, or their derivatives, which are limited to tantalum, tin, and tungsten’ – commonly referred to as ‘conflict minerals’ or ‘3TG’ – are covered by the legislation if the company suspects that they have originated in the DRC and bordering states. To take a typical such report, Lockheed Martin’s 2021 submission notes that 3TG ‘are in substantially all our products . . . for their functionality’ and that as a ‘downstream company’, establishing mineral provenance relies on the due diligence of a vast network ‘upstream’. In that year, Lockheed Martin’s 3TG were procured through 13,700 suppliers based in 55 countries.^18^ Raytheon’s 2022 submission reports 14,000 ‘globally dispersed’ suppliers with ‘multiple tiers . . . between RTC’s business segments and the 3TG mines . . . we rely on our first-tier suppliers to work with their upstream suppliers to provide us with accurate information . . . about the origin of 3TG contained in the materials we purchase.’^19^

At this stage we wish to justify something of a methodological conceit. We have drawn attention to cobalt, tantalum, copper and uranium not only because of their prominence in military technologies but also because we are able to approximate their provenance. The ‘conceit’ is justified thus: the anti-transparency of arms manufacturers, we reason, gives licence to make guided choices in analysis; it cannot be that the ‘closed doors’ of militarism close the doors of enquiry (see Belcher and Martin, 2013). Quite the opposite – they should instead prompt us to triangulate evidence so as to guide effective critique of the most profitable industry on the planet: war. We know that significant quantities of cobalt, copper, tantalum and uranium are mined in the DRC. That Lockheed Martin, Raytheon and other arms manufacturers effectively obfuscate the question of origin by passing responsibility ‘upstream’ adds substance to the licence we claim. We concede that we cannot know for certain that those and other companies source their raw materials from a nameable site, but we can learn from the conditions of extraction to know more about the origins of military violence. As might be clear at this point, our journey to these origins takes us from Iraq and Gaza to the DRC, where minerals are extracted that are then repackaged and deployed in the weaponry that distributes so much kinetic and ‘slow’ violence at sites of late modern war. It is here that we arrive at the extracted *geos*, the origins of the violent ends of war.

## The extractive origins of war

Around 3 km west of Kolwezi, the capital of Lualaba Province in the southern part of the DRC, the Musonoi mine reaches deep into the red earth. Covering a vast 25 km^2^, Musonoi is a particularly large example of similar sites across the DRC copperbelt, one of the world’s most mineral-rich geological formations that stretches through the south of the country and into northern Zambia. From above, the area, coincidentally or not, recalls a bombed-out landscape; the earth is pocked, displaced, discarded. Below ground lies 10% of the planet’s copper and more than a third of its cobalt, a ‘resource’ that attracts all types of mineral speculators, from multinational corporations to solo (or ‘artisanal’) miners and traders. There are tens of thousands of artisanal and company miners working at Musonoi, and there are many thousands more who depend on it in Kolwezi, where an economy of miners, middlepersons, security personnel, investors and support industries (hospitality, services, sex work) has grown. The term ‘artisanal miner’, for all its tones of craft and care, refers rather to miners who are not directly employed by mining companies. They are often poor migrant workers undertaking back-breaking labour in extremely dangerous conditions. There is little protective equipment, even as exposure to harmful materials – both the minerals themselves and the chemicals used to clean ores – is high, and there is a prevalence of respiratory problems, cancers and birth anomolies among miners and their dependents who live and work on the copperbelt (see e.g. Kayembe-Kitenge et al., 2019; Leon-Kabamba et al., 2018).

During a 2021–2022 field visit to mines in the Musonoi area where (mostly) cobalt and coltan (the metallic ore that contains niobium and tantalum) are extracted, Rubaii accompanied a Congolese research team from the University of Lubumbashi to conduct a study on miners’ health. In common with artisanal miners (*creuseurs*) across the copperbelt, those at Musonoi mine at their own risk and sell their products to distributors outside the mines. From these low-level traders, the cobalt and coltan moves through the supply chain via higher-level *négociants* (middlepersons) and *comptoirs* (buying houses) in larger cities such as Goma and Bukavu close to the Rwandan border. Ores then generally cross the border and travel to smelters, often in China, sometimes in Rwanda itself. The resulting products of cobalt, niobium and tantalum can then pass through multiple hands as they move ‘downstream’ to end-product tech companies such as Apple and Samsung, or Lockheed Martin and Raytheon. In 2022, 120,000 tonnes of cobalt (70% of global production) and 860 tonnes of tantalum (43% of global production) were produced through this route from the DRC and via a vast international network of smelters. This is a most convoluted supply chain where documentation is patchy with the effect that sure provenance is not easily determined.^20^ During onsite discussions with border-area ore traders, a point continually emphasized is that they rarely knew – *or indeed wished to know* – the precise mine from which commodities were sourced, and therefore it mattered little whether the minerals changing hands came from officially designated ‘conflict-free’ mines. What is more, traders make it their business *not to know*, creating a block in the flow of knowledge that, of course, downstream users readily identify as a basis for plausible deniability.

At the ‘upstream’ source of Musonoi, miners report a range of health effects. Around Kolwezi’s cafes and eateries, miners speak openly of exposure to coltan dust and uranium – the two ores occur together, making coltan potentially radioactive (Usanov et al., 2013) – and a frequency of breathing and chest problems during and after shifts underground. A recurring anecdote (told with some dark humour) is that some men are so irradiated that when they are close to a television the signal scrambles because of their static charge. The prevalence of this effect is unverified, but the claim in itself tells us that miners are aware of exposure and connect it to health. Other miners and their families report cancers and kidney problems, while accounts of children born with congenital anomalies are commonly shared. The issue has recently made mainstream press; the following is from a 2021 report in *The New Yorker*:

In Kolwezi, children as young as three learn to pick out the purest ore from rock slabs. Soon enough, they are lugging ore for adult *creuseurs*. Teen-age boys often work perilous shifts navigating rickety shafts. Near large mines, the prostitution of women and young girls is pervasive. Other women wash raw mining material, which is often full of toxic metals and, in some cases, mildly radioactive. If a pregnant woman works with such heavy metals as cobalt, it can increase her chances of having a stillbirth or a child with birth defects. (Niarchos, 2021)

There is a growing amount of research published in the medical sciences that concentrates on this last point: the long-term health effects of proximity with heavy metals in mining. A recent paper in *The Lancet*, for instance, details how artisanal miners in Kolwezi and other mines in southern DRC are exposed to high levels of cobalt, arsenic and uranium that has accumulated over decades of different processes of extraction (Van Brusselen et al., 2020). This is a causal factor, researchers argue, in ‘exposure-related oxidative DNA damage’ that indicates ‘associations between visible birth defects and paternal occupational mining exposure’ (Van Brusselen et al., 2020: 166). A paper in *Nature Sustainability* identifies links between the transformation of parts of Kolwezi into a cobalt mine, cobalt in blood samples, and DNA damage among workers and residents of the area (Banza Lubaba Nkulu et al., 2018). Further medical attention must be paid, the team of toxicologists argue, to how DNA changes might precipitate ‘a broad range of endpoints such as birth defects, neurodevelopmental impairment, respiratory disorders, heart and kidney disease, and cancer’ (Banza Lubaba Nkulu et al., 2018: 501; see also Leon-Kabamba et al., 2018).

Of deep concern in this longer-term view of mining and health is that not only are miners affected by exposure to toxic substances, so too are their families and (even unborn) children. But this is by no means the limits of harm imposed by mining activities: even the health of those with no direct connection to the mines is put at risk via a general degradation of the surrounding environment. The soils of Musonoi and around Kolwezi are depleted by deforestation, oily waterways weave through the mounds of displaced earth and stretch for kilometres, and there are few signs of the wildlife that once populated this part of central Africa. This eco-cide is owed to pollution from ever-increasing heavy machinery use, landfill and waste run off from a rapidly growing city (currently ~570,000 inhabitants), as well as the very serious issue of mining-related contaminants. Of these there are various discrete substances – e.g. acid mine drainage (ADM) from older or abandoned mines (Maheshe and Beak, 2018), naturally occurring radioactive materials (NORM) in coltan (Mustapha et al., 2007), fine cobalt particles that enter the food chain (Farjana et al., 2019: 150), and other mining effluents in the Musonoi river (Atibu et al., 2013) – that are brought into ecosystems when the earth is disturbed. The significance of these substances-made-contaminants is that the pathways of harm from mining activities are widened to entire communities who live on (and *off*) the land. One does not have to enter a mine shaft to be exposed, nor even to live with an exposed miner; mere proximity to sites of extraction can compromise health. There is an obvious parallel: just as the health of populations in Fallujah, Mosul and Gaza City is threatened by the war-damaged *geos*, so too is that of those of Kolwezi on the *geos* that constitutes war’s origins.

## Time, space, subjects

According to the evidence compiled so far, the toxic landscapes of war emerge both *before* and *after* the execution of formal military operations. This, we argue quite straightforwardly, sets war and *geos* in a doubly destructive relationship, leaving the question of how this notion affects our research practices and agenda. In this section we set out a framework for analysing *geos–*war relations and ecological beforemaths around three central parameters: spatiality, temporality, and the range of bodies affected.

In spatial terms, we are moved from the aeriality of war (drones, GPS, surveillance) to the ground below, and from the toxified soils of, for example, Iraq and Gaza to the deposits made toxic through extraction and removal in, for instance, the DRC. In her ethnography of the ‘front end of the nuclear cycle’ in Navajo Country, Traci Brynne Voyles (2015: 216) presents an instructive example of this extended geography where the uranium ‘gouged from the land by miners destined to suffer lung cancer . . . is then transformed into humanity’s most awesome and terrible weapons . . . deployed against yet more nations of colour and left to irradiate the desert lands of Iraq and Afghanistan’. Voyles renders explicit here a spatial connection between otherwise disparate beforemaths and aftermaths that also directs us to conceive the underground in a particular way: the subterranean is not only significant for bunkers and tunnels (e.g. Elden, 2013), nor for the seeping deposits of remnant munitions (Nixon, 2011; Pugliese, 2020), but also for the making of minerals from earth and the labouring bodies charged with extracting them. War in this sense does not only threaten the *geos* but derives from it; violence *on* the Earth is preceded by the question of violence *from* the Earth. Spatiality is also opened from much-discussed notions of remoteness – for instance, in the sustained (and important) attention brought to the notorious ‘drone vans’ in the Nevada desert (Gregory, 2011) – towards a proximity, or the body’s intimacy and contingency with the *geos* that premises the harms brought to miners and their communities.

In terms of temporality, the perspective on war we forward here guides us away from the currently prominent themes of speed and acceleration towards the antithetically paced processes of extraction, seeping, leaching, bleeding, mutation, gestation, metastasis, remission, relapse, and so forth. These processes are identified by medical professionals at the sites where war hardwares originate; it is remiss of us not to consider them in a fuller critique of war’s violence. Conversely, the idea of ‘slow violence’ must be critiqued by asking the simple but telling questions of ‘“slow” to whom? Whose gaze is privileged?’ (Cahill and Pain, 2019: 1058). Tumorigenesis and carcinogenesis can certainly be said to be ‘slower’ than a real-time targeting mission, but it serves to remember that ‘the deterioration of health . . . [occurs] quite rapidly from the most important perspective, that of one whose life is degraded by the ground made toxic by war’ (Griffiths, 2022: 292, 293). Finally on temporality, and as we have argued as the basis for this article, our recently broadened frame of time that extends ‘after’ war – for instance, in the idea of slower forms of violence – should also turn to the before, while all the time recognizing that even these temporal designations defer to a military’s formal declarations of the start and conclusion of operations. Where war’s violence begins, war begins – our analytical frames must reflect this.

This brings us to the range of bodies affected by war. Turning to the sites we do here vastly expands the subjects of potential future research and, importantly, refuses a militarized ontology of who is affected by war (see also Griffiths and Redwood, 2024). Existing categories currently foreclose the many groups involved in the extraction and production of military means of violence. For instance, ‘non-combatant’ or ‘quasi-combatant’ depend on the ongoing presence of a belligerent other, ‘collateral’ belongs only to the imaginaries of military administrations and arms manufacturers, and ‘civilian’ is reduced to the question of who is targetable under international law. Each of these examples comes into being only on the declaration of war, or the undertaking of military operations. Other categories – refugees, the injured and dead, veterans – render nameable certain other subjects of war in its aftermath; there are no counterpart figures available in our current ways of addressing war for those charged with extracting its materiality. How can an encephalopathic baby born to coltan miners be addressed as a subject of war? How are the patterns of sickness among miners themselves considered a part of war’s injured? And, importantly, how are the racialized logics of war targeting threaded through the ecological beforemaths of advanced military technologies? In response, we should first acknowledge that we have not yet formulated an address of those lives (and deaths) that are intimately tied to the war-affected *geos*; where weapons visit harm not through kinetics but as toxic materialities, our idea of ‘injury’ is insufficient to capture anything of the epidemiologies of ‘pre’-war populations. Nor have we yet fully realized the racializing connections (e.g. identified by Voyles above) between indigeneity/coloniality, bombing and extraction in discussions of war and militarism. As technologies of war further advance and proliferate, it is an urgent project to bring these increasingly crucial groups into view as subjects of war’s violent effects.

To the *geos* itself, where it all begins. We are of course not the first to argue that the environment be considered a primary subject of war, but the evidence here indicates a need to set the Earth and its elements as central to the doing and effects of war. This is where the term *geos* gains critical purchase by backgrounding the narrowly geopolitical categories of ‘zone’ or ‘territory’ and, as Elizabeth Povinelli (2016, 2017) has so clearly written, by foregrounding a porosity between ‘life’ and ‘non-life’ boundaries in the earth. In this ontology, the Earth’s constitutive elements sustain, give and take life; harm to one is harm to another; and the land is a primary referent from which we are not, despite all the machinations of industrialized society, ever entirely separated. The *geos* can thus be conceived as that which both precedes and sustains categories of political (*bios*) and biologized (*zoē*) life, it is co-extensive with the Earth (as land, sea, air) while also predicating an ontological field of plant and non-plant life (insofar as plant/non-plant distinctions hold) (see also Griffiths, 2022; Johnson et al., 2019). The critical utility of *geos* is realized, to return to the case to hand, in learning from the epidemiologists, doctors, public health specialists, miners and their families cited above. The ground cannot be reduced to a ‘zone’ in which war takes place or a capturable ‘territory’ that drives war, nor can it be parsed as ‘commodity’ for mineral extraction that is entirely distinct from the extracting body. The *geos* is the analytical key to a clearer view of the Earth and its elements that are set to work in the distribution of military violence, from extraction in the beforemath to deposits in the aftermath. Spatial connections between mineral-rich areas, the makers of war (US, UK, Israel, coalition states), and their targets (e.g. Afghanistan, Gaza, Iraq) can thus be mapped alongside the specific actors who put the *geos* to violent use (mining companies, arms manufacturers) and whose lives are lived in and through the *geos*-made-toxic.

The notion of *geos* thus takes us from aftermaths to beforemaths in a way that challenges us to see neither as addenda to war but rather as integral to it, with the critical political assertion that there are no ‘indirect’ or ‘collateral’ consequences; both *before* and *after* are constitutive of war itself. War begins and ends in the *geos*.

## Conclusion

Late modern war is a form of asymmetric, technologized warfare that produces devastating aftermaths in which war’s violence endures for years and generations. This consists in the destruction of infrastructure, the destabilizing of political regimes, and, as we have focused on in this article, the instrumentalized and damaged environment – or *geos* – that exposes populations to long-term harm. While scholarly attention has grown around such aftermaths, we are yet to ask important questions about antecedents, or what we refer to as the ‘beforemath’. We advocate for further research on war’s beforemaths and set out how this might bring us to new understandings of war in terms of spatiality, temporality and the range of bodies affected. By way of conclusion, we wish to explicate three points that emphasize the political urgency of turning critical attention to a notion of a doubly destructive relationship between war and *geos.*

First, while tech companies such as Apple, Samsung and Tesla are routinely cited in critiques of the mining industry (especially in the DRC), there is scarce mention of weapons manufacturers, even as they (Lockheed Martin and Raytheon are just two examples) use vast quantities of technologically critical elements. The secretive and uneven relations that constitute the militarized geopolitics of supply chains (see e.g. Cowen, 2014; Khalili, 2021) should not excuse such manufacturers from implication in the devastation visited on mining communities and their lands.^21^ Second, and connectedly, the approach to war we develop here threatens to open new avenues of political accountability. Against the state-protected secrecy around weapons production, the massive growth of the industry has coincided with a rhizomatic expansion of supply chains that are, inevitably, uneven in terms of data protection, liability to public record, non-disclosure protocols and investor reporting. The political repercussions of this are already felt where, for instance, pro-Palestine activists have disrupted and closed down facilities in the UK that were operated by ‘upstream’ supplier-subsidiaries of one of Israel’s largest weapons manufacturers, Elbit Systems^22^ – a task that is made even more urgent during the ongoing (at time of writing) military assault on Gaza in 2023–2024. A political opening from here might develop a public discourse on the complicity of traders and smelters, as well as mining corporations (e.g. Eurasian Resources Group, Adastra and Glencore are each on record as working at or around Musonoi) which profit at the source of war’s harmful materialities.

Third, and finally, we only scratch the surface here. The examples of the invasion of Iraq and Operation Cast Lead in Gaza, while devastating in themselves, are just two discrete military assaults of many. The current military operations in Gaza have already (as of March 2024) used the arsenal of Operation Cast Lead many times over, as has Russia’s invasion of Ukraine. A fuller account of the doubly destructive *geos–*war relationship would take in not only those many other acts of war but also the vast minerals put into prototypes, testing, marketing, transporting and deploying the gruesome weapons that are fashioned from the extracted *geos.* In this way we can add further political vitality to the idea of ‘conflict minerals’ and the ways that late modern war’s relationship with the *geos* is *doubly* destructive.

## Notes

1. This article was written before Israel’s military invasion of Gaza after the Hamas attacks on 7 October 2023.

2. On the politics of research into congenital disorders in Iraq see The Nation (2020)

3.
https://www.iraqbodycount.org/

4. RAND Corporation (Johnson, 2011: 114) calculates higher numbers: ‘during the course of the operation, the IAF flew 5,650 sorties for a total of 20,650 flight hours. There were 1,700 fixed-wing attacks and 1,150 attack-helicopter attacks against a total of 3,430 targets. Of the 5,500 munitions employed, 81 per- cent were precision guided.’

5. These weapons are ‘suspected’ to have been used (Esposito, 2009b: 184–189).

6. Crawford (2022: 6) makes the following ‘conservative’ calculation: ‘U.S. military and military industry emissions for the top twelve U.S. military manufacturers totalled 105.7 MMTCO_2_e in 2019 . . . about equal to the combined emissions of the world’s forty-five smallest emitting countries.’

7.
https://metals.wallcolmonoy.com/f-16-military-aircraft-heat-exchangers

8.
https://www.stanfordmagnets.com/samarium-cobalt-magnets-in-the-military.html

9.
https://www.darpa.mil/about-us/timeline/rare-earth

10.
https://www.cbsnews.com/news/pilotless-warriors-soar-to-success/

11.
http://www.strongtantalum.com/tag/tantalum-alloy/

12.
https://www.af.mil/News/Article-Display/Article/139480/b-52-litening-ii-pod-used-in-combat/

13.
https://www.northropgrumman.com/what-we-do/air/litening-advanced-targeting-pod/

14. Testimony of Lieutenant General Edward Hanlon (US Marine Corps) before the House Armed Services Committee Subcommittee on Tactical and Land Forces US House of Representatives: https://www.globalsecurity.org/military/library/congress/2004_hr/04-04-01hanlon.htm?utm_content=cmp-true

15.
https://www.f-16.net/f-16_armament_article5.html

16.
https://www.japcc.org/wp-content/uploads/JAPCC_UAV_Flight_Plan_2008_screen.pdf

17.
https://armamentresearch.com/wp-content/uploads/2021/10/ARES-Special-Report-No.-4-Munitions-Employing-Sensor-fuzedSubmunitions.pdf

18.
https://www.lockheedmartin.com/content/dam/lockheed-martin/eo/documents/suppliers/conflict-minerals-cmr-2021.pdf

19.
https://investors.rtx.com/static-files/765e55a0-fe27-4a6c-b5ac-649cf6fa71cc

20. For a detailed account of the supply chain see Smith (2022: 105–156)

21. Writing at an early stage of advanced technologies in war, Biswas (2000: 306–307) claims that ‘overall, on a global basis, between 2 and 11 percent of fourteen important minerals is consumed for military purposes: aluminium, chromium, copper, fluorspar, iron ore, lead, manganese, mercury, nickel, and platinum for military purposes is greater than Latin America’s consumption for all purposes. The environmental impacts of the mining activities associated with such high levels of extraction of resources are certainly significant.’

22.
https://www.theguardian.com/world/2017/nov/23/charges-dropped-protest-israeli-military-drones-factory-uk-uav-engines
